# CRISPR-Cas9-Mediated Mutation of Methyltransferase METTL4 Results in Embryonic Defects in Silkworm *Bombyx mori*

**DOI:** 10.3390/ijms24043468

**Published:** 2023-02-09

**Authors:** Hao Guo, Feng Chen, Mingyi Zhou, Weiqun Lan, Wenchang Zhang, Guanwang Shen, Ping Lin, Qingyou Xia, Ping Zhao, Zhiqing Li

**Affiliations:** 1 State Key Laboratory of Silkworm Genome Biology, Biological Science Research Center, Southwest University, Chongqing 400715, China; 2 Integrative Science Center of Germplasm Creation in Western China (CHONGQING) Science City & Southwest University, Chongqing 400715, China; 3 Chongqing Key Laboratory of Sericultural Science, Chongqing Engineering and Technology Research Center for Novel Silk Materials, Southwest University, Chongqing 400715, China

**Keywords:** *Bombyx mori*, CRISPR-Cas9, METTL4, N6-methyladenine, embryonic development

## Abstract

DNA N6-methyladenine (6mA) has recently been found to play regulatory roles in gene expression that links to various biological processes in eukaryotic species. The functional identification of 6mA methyltransferase will be important for understanding the underlying molecular mechanism of epigenetic 6mA methylation. It has been reported that the methyltransferase METTL4 can catalyze the methylation of 6mA; however, the function of METTL4 remains largely unknown. In this study, we aim to investigate the role of the *Bombyx mori* homolog METTL4 (BmMETTL4) in silkworm, a lepidopteran model insect. By using CRISPR-Cas9 system, we somatically mutated BmMETTL4 in silkworm individuates and found that disruption of BmMETTL4 caused the developmental defect of late silkworm embryo and subsequent lethality. We performed RNA-Seq and identified that there were 3192 differentially expressed genes in BmMETTL4 mutant including 1743 up-regulated and 1449 down-regulated. Gene Ontology and Kyoto Encyclopedia of Genes and Genomes analyses showed that genes involved in molecular structure, chitin binding, and serine hydrolase activity were significantly affected by BmMETTL4 mutation. We further found that the expression of cuticular protein genes and collagens were clearly decreased while collagenases were highly increased, which had great contributions to the abnormal embryo and decreased hatchability of silkworm. Taken together, these results demonstrated a critical role of 6mA methyltransferase BmMETTL4 in regulating embryonic development of silkworm.

## 1. Introduction

DNA modification by methylation of adenine residues at the N6 position—to generate N6-methyladenine (6mA)—has been identified as the most abundant internal chemical modification in prokaryotes, which plays crucial roles in DNA replication and repair, transcription, and cell cycle regulation [1,2,3,4,5]. Recently, several studies have reported the presence of 6mA in genomic DNA from multicellular eukaryotes, such as *Caenorhabditis elegans*, *Drosophila melanogaster*, *Arabidopsis thaliana*, *Oryza sativa*, *Xenopus laevis*, *Mus musculus*, *Danio rerio*, and *Homo sapiens* [6,7,8,9,10,11,12]. Although the emerging biological roles of 6mA in eukaryotes have reported to be involved in gene expression, chromatin regulation, stress response, and human cancer progression and tumorigenesis [13,14,15,16], the low abundance of 6 mA methylation in distinct eukaryotes have limited the research. Moreover, the functions of 6mA methylation in eukaryotes are still largely unknown compared to those in prokaryotes.

By using various sequencing strategies, more and more studies have determined the genomic location of 6mA in a genome-wide profile in eukaryotic organisms, which establishes the association of 6mA roles with gene expression, cell cycle regulation, nucleosome positioning, and chromatin regulation [16,17]. However, the evidence to elucidate direct functions of 6mA methylation still needs to be uncovered. It has been reported that the methylation of 6mA is dynamically regulated by methyltransferase and demethylase [6,7]. The homology search for 6mA methyltransferases in eukaryotes has revealed that the MT-A70 domain evolved from the classic bacterial M.MunI would be the putative 6mA methyltransferase in eukaryotes [18]. For instance, DAMT-1 in *C*. *elegans* and N6AMT1/METTL4 in mammals have been identified as 6mA methyltransferases [6,11,13]. In regard to demethylation of 6mA, it has been shown that the demethylase belong to the ALKB family, which is conserved from bacteria to humans [16,19]. In humans, ALKBH1 and ALKBH4 have been proposed to demethylate 6mA and its homologue of NMAD-1 in *C*. *elegans*. Furthermore, the human TET1 homologue of DMAD in *D*. *melanogaster* has also been reported as DNA 6mA demethylases [6,7,20,21]. However, the biological roles of these methyltransferase and demethylase-mediated 6mA methylation and demethylation in eukaryotes were largely unknown.

In *D*. *melanogaster*, loss of DMAD significantly increased the levels of 6mA methylation, and further demonstrated its regulatory role in *D*. *melanogaster* development and early germ cell differentiation [7]. In *C*. *elegans*, deletion of NMAD-1 promoted the progressive fertility defect phenotype of spr-5 mutant worms, while this defect phenotype could be suppressed by DAMT-1 deletion due to the reduced 6mA levels [6]. In human cancer cells, the silencing of N6AMT1 decreased 6mA levels, promoted cancer cell growth, colony formation, migration, and invasion, whereas ALKBH1 silencing inhibited cancer cell growth, colony formation, migration, and invasion, which suggested that the decrease of 6mA levels promoted tumorigenesis [11]. Only a limited number of studies so far have reported the functions of 6mA methyltransferase and demethylase that show quite distinct roles in different species.

Searching for specific 6mA methyltransferase and demethylase is important for understanding the potential role of 6mA in other eukaryotes. In the previous study, we have identified the methyltransferase BmMETTL4 and demethylase BmNMAD in lepidopteran model silkworm *Bombyx mori*, and furthermore, demonstrated their catalyzed activities on 6mA methylation, regulatory roles in chromosome congression, and segregation in cultured silkworm cells [22]. In the current study to further elucidate the physiological function of the methyltransferase BmMETTL4 in individual development of silkworm, we somatically mutated BmMETTL4 by using a transgenic clustered regularly interspaced short palindromic repeat/CRISPR-associated protein 9 (CRISPR-Cas9) system [23,24]. We found that mutation of BmMETTL4 caused the developmental defect of late silkworm embryo and led to the failure of hatching. Further RNA-Seq and differentially expressed gene (DEG) analyses revealed that many different Gene Ontology (GO) functions and Kyoto Encyclopedia of Genes and Genomes (KEGG) pathways were regulated by BmMETTL4. These analyses also revealed a large number of structural constituents were significantly down-regulated in BmMETTL4 mutant, which may result in abnormal embryonic heads and thus affect the hatchability of silkworm. Altogether, our present data revealed a crucial function of 6mA methyltransferase BmMETTL4 in regulating embryonic development of silkworm.

## 2. Results

### 2.1. CRISPR-Cas9 System Induced the Mutation of BmMETTL4

**Figure 1:**
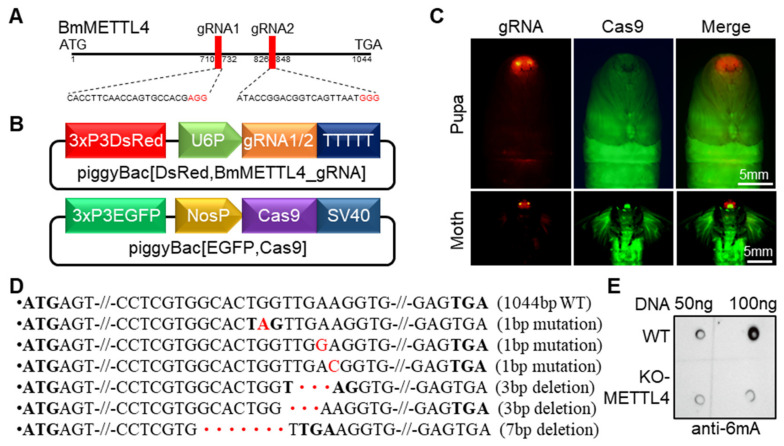
CRISPR-Cas9 system induced the mutation of BmMETTL4. (**A**) A schematic diagram of the BmMETTL4 gene structure and gRNA-target sites. (**B**) Plasmid construction for generation of the transgenic silkworm. (**C**) Positive transgenic silkworms co-expressing gRNAs and Cas9. The positive silkworm pupa and moth were screened under RFP fluorescence (red) and GFP fluorescence (green), respectively. Scale bar is 5 mm. (**D**) PCR-based amplification and sequencing of regions targeted by gRNAs in KO-METTL4 and WT silkworms. The nucleotides labeled in red were replacement, the dots in red were depleted nucleotides, and the bold nucleotides were stop codon. (**E**) Genomic DNAs isolated from the embryos of KO-METTL4 and WT silkworms were subjected to dot blot assays.

It has previously been reported that the sole DNA 6mA methyltransferase BmMETTL4 in silkworm was involved in the methylation of N6-adenine and possessed dynamic expression during embryogenesis of silkworm [22]. In order to decipher the regulatory roles of BmMETTL4-mediated 6mA methylation on development of silkworm, we used a transgenic CRISPR-Cas9 system to generate BmMETTL4 mutant, as previously described [24]. We designed two guide RNA (gRNA) target sites in open reading frame (ORF) of BmMETTL4 (Figure 1A) and constructed a gRNA expression vector under the control of the silkworm U6 promoter with DsRed selective marker (Figure 1B). Meanwhile, the expression of Cas9 was driven by the silkworm Nanos promoter with EGFP selective marker (Figure 1B). After the hybridization of these two silkworm strains, the offspring expressed by both gRNAs and Cas9 were selected and used to analyze the editing profiles (Figure 1C). It was shown that the somatic mutations were detected by PCR sequencing using gene-specific primers, which indicated that KO-METTL4 mutant had generated various mutations and deletions (Figure 1D and Table S1). Because of the great changes of 7 bp deletion in both nucleotides and amino acids, we subsequently maintained this KO-METTL4 mutant for further analysis. To evaluate the effects of BmMETTL4 mutant on the 6mA modification, we analyzed the levels of 6mA methylation in KO-METTL4. As shown in Figure 1E, the dot blot assay showed that 6mA levels in KO-METTL4 were much lower than WT. Considered together, these data demonstrated that the transgenic CRISPR-Cas9 system can effectively disrupt the BmMETTL4 gene in silkworm.

### 2.2. Depletion of BmMETTL4 Caused Developmental Defect of Silkworm Embryo

**Figure 2:**
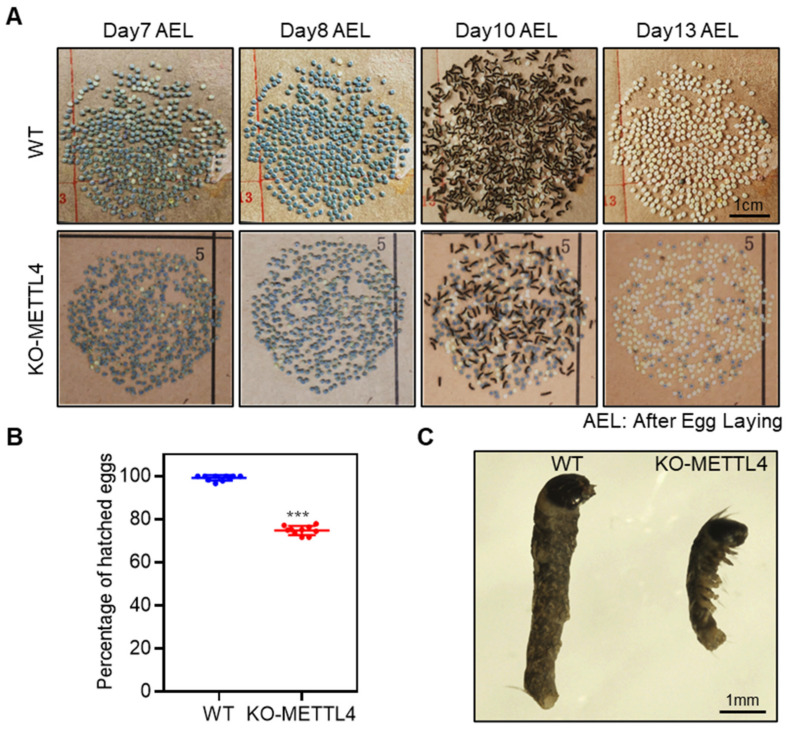
Depletion of BmMETTL4 caused developmental defect of silkworm embryo. (**A**) Time-lapse of embryonic development in KO-METTL4 and WT silkworms. Scale bar is 1 cm. (**B**) Percentage of hatched eggs that were laid from KO-METTL4 and WT silkworms. *** *p* < 0.001. (**C**) Representative images of the internal embryos of WT and KO-METTL4 dissected from eggs before hatching. Scale bar is 1 mm.

After the crossing of BmMETTL4 mutant strains, it was observed that eggs laid from KO-METTL4 strain were frequently interrupted at the last stage of embryonic development that cannot be hatched normally. To examine the detailed changes of embryos, we traced the developmental process of eggs during the last few days. The embryos in the eggs from KO-METTL4 could normally proceed to the stage of serosa and abdominal pigmentation in which almost all the silkworm eggs have turned colors and reached the stage close to hatching on day 8 after oviposition (Figure 2A). However, as all WT silkworms were hatched on day 10 after oviposition, only a small number of embryos of KO-METTL4 could develop into larvae and the remaining embryos in the eggs did not further hatch even on day 13 after oviposition (Figure 2A). We analyzed the hatchability of WT and KO-METTL4, which showed that more than 25% of embryos from KO-METTL4 had died (Figure 2B). To understand the phenotypic consequence of these abnormal embryos, we dissected the unhatched embryos from eggs. It was clearly shown that the embryos of KO-METTL4 were much smaller than that of WT silkworms (Figure 2C). These results indicated that BmMETTL4 mutant affected the development of the late silkworm embryo and led to the failure of hatching.

### 2.3. Depletion of BmMETTL4 Led to Great Changes in Transcriptome

**Figure 3:**
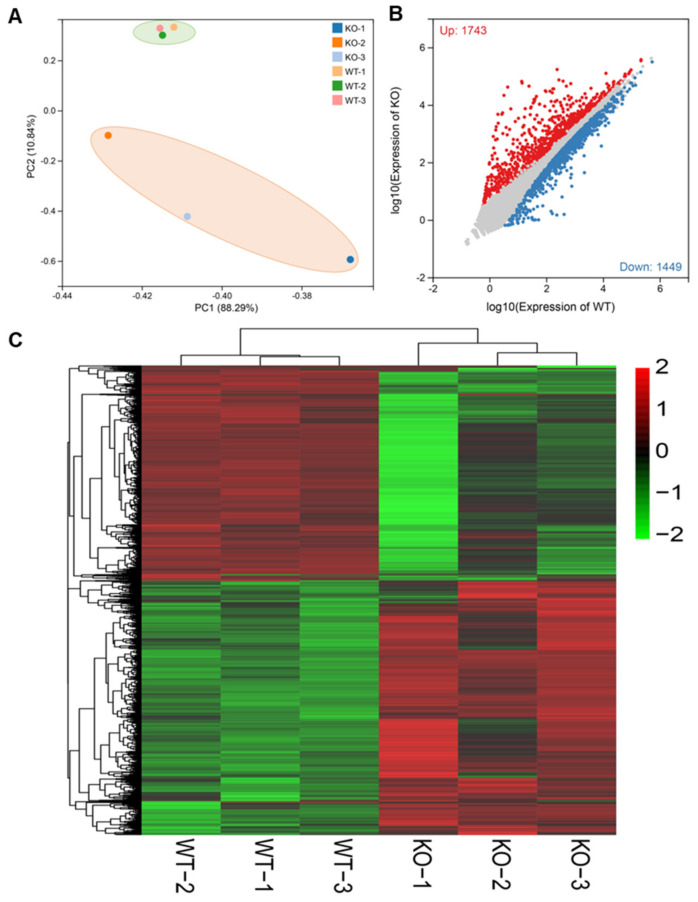
Depletion of BmMETTL4 led to great changes in transcriptome. (**A**) PCA of transcriptome data among different samples were analyzed by plotPCA function of DEseq2 program. (**B**) Statistical chart of DEGs in WT and KO-METTL4 mutant. The standard |log2(Fold Change)| > 0 and *Padj* < 0.05 were used to determine significant differences in gene expression. Red represents up-regulated genes, blue represents down-regulated genes, and gray represents genes without significant differences. (**C**) Heatmap visualization of DEGs between WT and KO-METTL4 mutant.

To explore the molecular mechanisms of BmMETTL4-mediated regulation on embryonic development, we collected the unhatched embryos, in which the egg shells were removed from KO-METTL4, and hatched silkworms from WT for comparative transcriptional analysis by RNA-Seq. Principal component analysis (PCA) of the KO-METTL4 and WT demonstrated that all samples were clearly divided into two distinct groups (Figure 3A). After comparing the transcriptional data of the KO-METTL4 and WT, a total of 3192 DEGs were identified (Tables S4 and S5). Among these genes, 1743 were up-regulated and 1449 were down-regulated in the KO-METTL4 mutant compared to the WT (Figure 3B). Furthermore, a general overview of the expression profile was visualized by a heatmap (Figure 3C), illustrating a significant expression difference after depletion of BmMETTL4 and providing an overall understanding of DEG expression.

**Figure 4:**
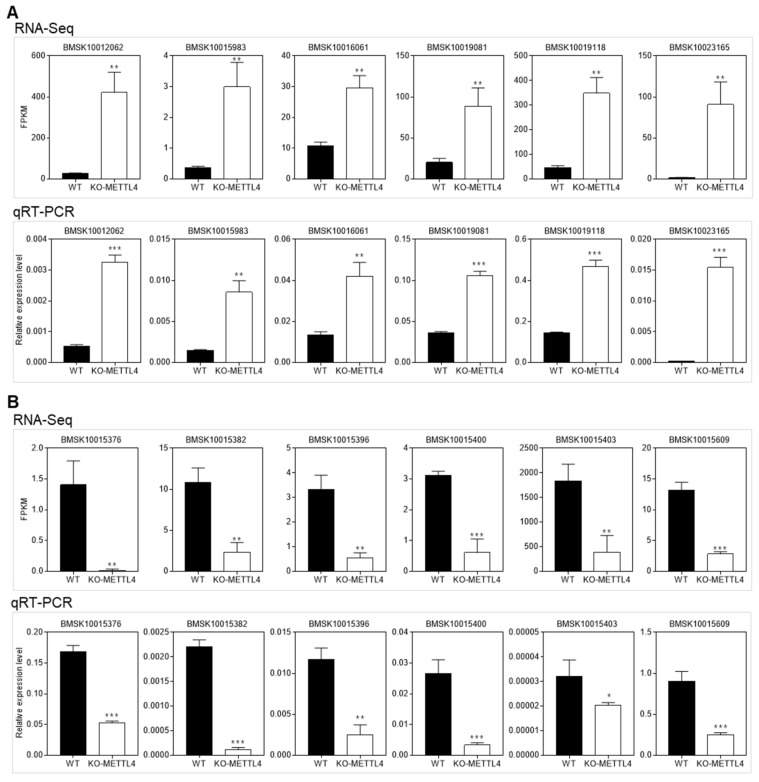
qRT-PCR verification of the RNA-Seq data. (**A**) qRT-PCR was used to examine the up-regulated DEGs from RNA-Seq. (**B**) qRT-PCR was used to examine the down-regulated DEGs from RNA-Seq. Each sample was repeated in triplicate and all data were presented as the mean ± standard deviation (SD). A student’s t-test was used to evaluate statistical significance (* *p* < 0.05, ** *p* <0.01, and *** *p* < 0.001).

To validate the RNA-Seq data, we randomly selected 12 DGEs from up-regulated and down-regulated genes for qRT-PCR analysis. It was shown that the qRT-PCR results were consistent with the transcriptome data (Figure 4), which further confirmed the reliability of the RNA-Seq data.

### 2.4. Depletion of BmMETTL4 Induced Differential GO and KEGG between Up-DEGs and Down-DEGs

**Figure 5:**
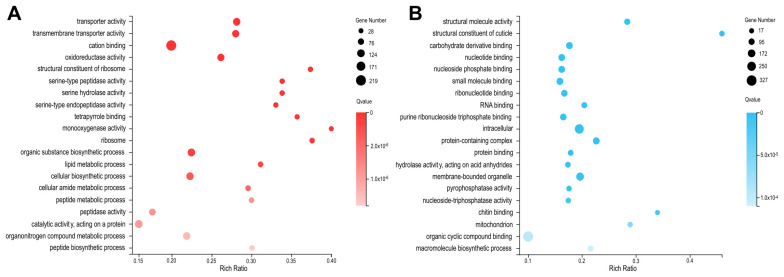
GO annotations of DEGs between WT and KO-METTL4 mutant. Functional categories of DEGs were analyzed by Gene Ontology (GO). The images exhibited top20 statistics of up-DEGs (**A**) and down-DEGs (**B**) on GO annotations.

To understand the gene functions related with depletion of BmMETTL4 in embryonic development, we analyzed the functional annotations by GO terms in up-DEGs and down-DEGs, respectively. The top 20 statistics of GO annotations were listed in Figure 5. It was shown that the up-regulated DEGs in BmMETTL4 mutant were mainly enriched in monooxygenase activity, structural constituent of ribosome, tetrapyrrole binding, and serine hydrolase activity (Figure 5A), whereas the down-regulated DEGs were involved in structural constituent of cuticle, chitin binding, molecular structure activity, and mitochondrion (Figure 5B).

**Figure 6:**
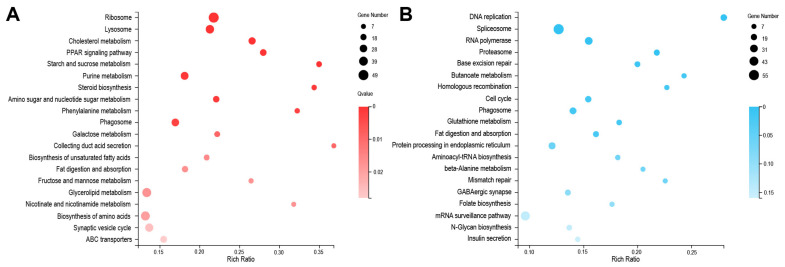
KEGG pathways of DEGs between WT and KO-METTL4 mutant. Signal pathway enrichments of DEGs were analyzed by Kyoto Encyclopedia of Genes and Genomes (KEGG). The images exhibited top 20 statistics of up-DEGs (**A**) and down-DEGs (**B**) on KEGG pathways.

To further gain insight into the biological pathways after BmMETTL4 depletion, DEGs were mapped to the KEGG database and the top 20 enrichments of up-DEGs and down-DEGs were shown in Figure 6. The pathways of up-regulated DEGs in BmMETTL4 mutant were significantly enriched in collecting duct acid secretion, starch and sucrose metabolism, steroid biosynthesis, and phenylalanine metabolism (Figure 6A). For the down-regulated DEGs, the significantly enriched KEGG pathways were DNA replication, butanoate metabolism, homologous recombination, and mismatch repair (Figure 6B). The differential GO and KEGG between up-DEGs and down-DEGs indicated the crucial roles of BmMETTL4 during silkworm embryogenesis

### 2.5. Down-Regulated Structural Constituents in BmMETTL4 Mutant May Result in Abnormal Silkworm Embryo

**Figure 7:**
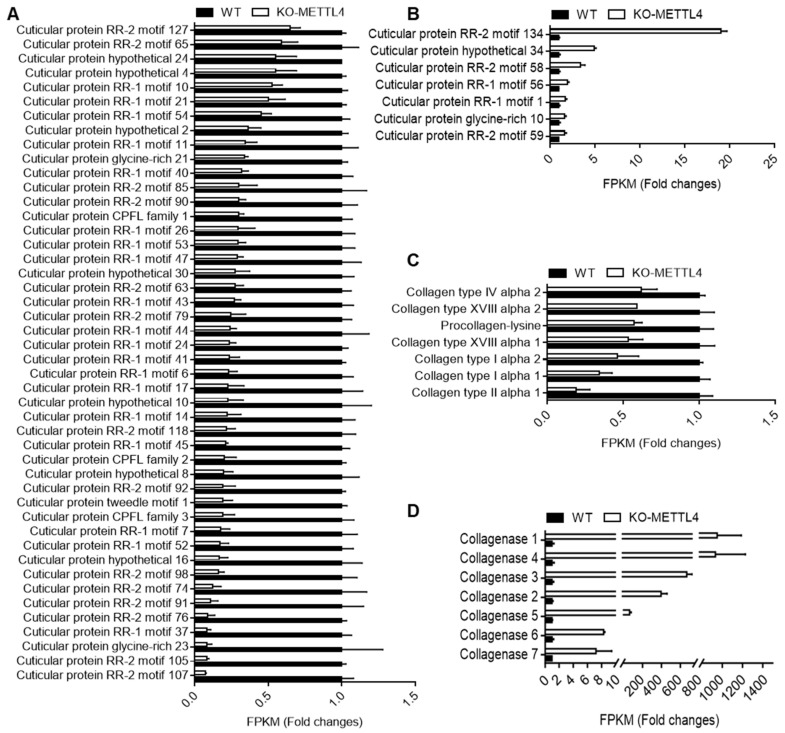
Expression alteration of main structure protein genes. (**A**) Down-regulated cuticular protein genes in KO-METTL4 mutant. (**B**) Up-regulated cuticular protein genes in KO-METTL4 mutant. (**C**) Down-regulated collagens in KO-METTL4 mutant. (**D**) Up-regulated collagenases in KO-METTL4 mutant. The FPKM value in WT was set as 1 and the fold changes of FPKM value in KO-METTL4 after normalization to WT were shown.

From the GO and KEGG analyses of DEGs, we found that the structural constituent of cuticle was significantly enriched in down-regulated DEGs. We therefore surveyed all the cuticular protein genes in DEGs between the KO-METTL4 mutant and WT. It was clearly shown that 46 cuticular protein genes were significantly down-regulated in KO-METTL4 (Figure 7A) and only 7 genes were up-regulated (Figure 7B). Moreover, these cuticular protein genes had covered almost all the cuticular protein families of RR-1, RR-2, Tweedle, CPFL, CPG, and CPH, with the exception of RR-3 [25]. Intriguingly, another group of molecular structure such as collagens were all down-regulated in mutation of BmMETTL4 (Figure 7C), whereas collagenase responsible for degrading native collagen were significantly up-regulated (Figure 7D) [26,27]. These analyses indicated important roles of structural proteins in the process of embryogenesis.

**Figure 8:**
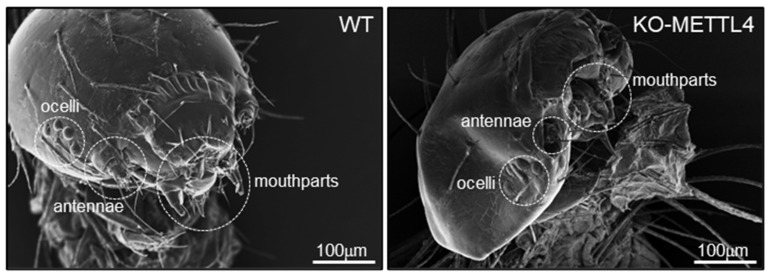
Morphological observation of the silkworm head in the WT and KO-METTL4 mutant. Representative images of the silkworm head of the WT and KO-METTL4 mutant as examined by SEM microscope. The structures of mouthparts, antennae, and ocelli were labeled by broken circles. Scale bar is 100 μm.

Due to the failure of hatching of BmMETTL4 mutant silkworm, we speculated that the large number of cuticular protein genes and collagens down-regulated by the depletion of BmMETTL4 may affect the formation of hard cuticle and normal morphology of silkworm embryo. To examine this possibility, we dissected the late embryos of silkworm from the KO-METTL4 and WT, and screened the heads under a SEM. As shown in Figure 8, we observed a morphological abnormality of the head in KO-METTL4 embryos and numerous defects in head shapes and structures, especially on the mouthparts, antennae, and ocelli that were seriously atrophied compared to the WT. These data suggested that the majority of down-regulated structural constituents in BmMETTL4 mutant probably resulted in developmental defects of silkworm embryo and thus affected the hatchability of silkworm.

## 3. Discussion

As a major epigenetic modification, DNA N6-adenine methylation is widespread in prokaryotes and only recent studies have uncovered the genome-wide distribution and potential function of 6mA in several eukaryotes [16,17]. However, due to the low abundance of 6mA levels in eukaryotes and the lack of biochemical evidence for methyltransferase and demethylase in catalyzing 6mA, the roles of 6mA methylation and enzymes in eukaryotes remains to be further elucidated. It has been reported that the putative 6mA methyltransferase DAMT-1 in *C*. *elegans* was involved in progressive fertility of worms [6]; METTL4 in mouse 3T3-L1 cells promoted adipocyte differentiation [28]; METTL4 in humans contributed to mitochondrial DNA 6mA methylation; [13] and also mediated m6Am methylation of U2 small nuclear RNA (snRNA) [29]; while METTL4 in *D*. *melanogaster* catalyzed U2 snRNA m6A methylation had broad impacts on various biological pathways through altered RNA splicing [30]. In addition to METTL4 in *D*. *melanogaster*, the role of METTL4 in other insects remain largely unknown. In this study, we therefore investigated the role of BmMETTL4 in the development process of silkworm. By using CRISPR-Cas9 system-mediated mutation of BmMETTL4, we demonstrated that BmMETTL4 depletion severely influences silkworm embryonic development, which resulted in embryonic lethality at the late stage of embryonic development.

A previous study reported that DNA 6mA methylation was widely distributed during embryogenesis of silkworm and that the methyltransferase of BmMETTL4 was dynamically expressed throughout the embryonic development, which suggested a critical role of BmMETTL4-mediated 6mA methylation in silkworm embryo [22]. Indeed, depletion of BmMETTL4 not only led to decreased 6mA levels, but also induced severe defects in embryonic development. To gain insight into the regulatory role of BmMETTL4, we carried out transcriptome analysis and demonstrated that BmMETTL4 mutation altered numerous gene expressions. It showed a significant up-regulation of DEGs that were functionally related with structural constituent of ribosome, serine hydrolase activity, and steroid biosynthesis. Meanwhile, down-regulation of DEGs were functionally enriched in structural constituent of cuticle, chitin binding, and molecular structure activity. These analyses indicated the crucial roles of BmMETTL4 in regulating embryonic development of silkworm.

Interestingly, it was discovered that a large number of cuticular protein genes were significantly down-regulated by depletion of BmMETTL4. In insects, the cuticle is produced by the epidermis and mainly composed of chitin together with many kinds of cuticular proteins [25]. Insect cuticle will protect the body during the whole life cycle from the damage of the external environment. As for insect eggs, the extraembryonic serosa will form a serosal cuticle that protects the embryo from desiccation and infection during embryonic development [31,32]. The absence of the cuticular proteins and chitin synthesis led to the defective embryogenesis and even lethality in several insects [33]. Therefore, the down-regulated cuticular protein genes in BmMETTL4 mutant may result in abnormal formation of cuticle in embryonic head that causes numerous defects in mouthparts, antennae, and ocelli of silkworm. The further knockout of these cuticular protein genes in silkworm could be done to confirm the present observation, which will provide novel contribution of cuticular proteins in the formation of embryonic cuticle in insects.

We also found that there were many DEGs possessing molecular structure activity, including structural molecular proteins in the extracellular matrix (ECM)-interaction pathway such as collagens, that were all generally down-regulated in BmMETTL4 mutant. Collagen can provide structural support for cell growth and proliferation, help maintain the structural integrity of the body and organs, and play a crucial role during embryonic tissue morphogenesis [26]. In silkworm, we identified 17 collagens belonging to different collagen types in the genome, of which 7 collagens were significantly repressed by depletion of BmMETTL4. In contrast, there were no collagens in the up-regulated DEGs, suggesting the synergic action of collagens in embryonic morphogenesis of silkworm. Alternatively, it was found that there were 7 collagenases highly expressed in BmMETTL4 mutant. Collagenases are enzymes with serine hydrolase activity, which are capable of degrading native collagen under physiological conditions [27]. The significant up-regulation of these enzymes would further destroy the molecular structure activity of collagens. Thus, mutation of BmMETTL4 for small body and decreased hatchability of silkworm embryo was possibly associated with the influence of the balance between collagens and collagenases, and finally, contributed to the defects of embryonic development.

The methyltransferase METTL4 is widespread and plays critical roles in methylating genomic DNA, mitochondrial DNA, and U2 snRNA, and thus regulates a wide range of gene expressions [13,28,29,30]. The present work has identified potential target genes of BmMETTL4 during silkworm embryonic development. It was speculated that these target genes would be regulated by BmMETTL4-mediated DNA 6mA methylation. Although highly expressed genes were usually found to be associated with increased 6mA methylation, the methylation levels on different gene targets also showed distinct effects on gene transcriptions, which implicated a complicated 6mA methylation regulation on the specific genes [16,28]. Therefore, further experiments regarding the genome-wide mapping of 6mA methylation status between BmMETTL4 mutant and WT will give us clues on how 6mA methylation regulates expression of these target genes.

In summary, our data provided the first evidence that BmMETTL4 plays a crucial role in regulating silkworm embryonic development. We speculated that BmMETTL4-mediated 6mA methylation would regulate the expression of numerous target genes in silkworm. Significantly, the down-regulated genes associated with cuticular protein genes and collagens, and to a larger extent, the up-regulated genes related to collagenases, will contribute to the abnormal embryo and result in decreased hatchability of silkworm. On the other hand, BmMETTL4-mediated 6mA methylation should be vital for the embryonic development in silkworm. Further functional research on 6mA methylation and target gene expression will provide regulatory mechanism of BmMETTL4-mediated 6mA methylation during silkworm embryogenesis.

## 4. Materials and Methods

### 4.1. Silkworm Strain

The multivoltine non-diapausing strains D9L and Nistari-Nos-Cas9 transgenic silkworms were maintained in our laboratory [34]. All silkworm larvae were fed by fresh mulberry leaves or artificial diet under standard conditions.

### 4.2. Plasmid Construction

The guide RNAs (gRNAs) were designed for the exon of BmMETTL4 by using CCTop CRISPR-Cas9 target online predictor [35]. All gRNAs and primer sequences for plasmid construction were listed in Table S1. The plasmid piggyBac[DsRed,BmMETTL4_gRNA] were constructed to express gRNA under the control of the silkworm U6 promoter and the DsRed fluorescence marker gene under the control of an IE1 promoter. The plasmids were verified by sequencing (BGI, Shenzhen, China).

### 4.3. Germline Transformation

Generation of transgenic silkworms were performed according to a previously reported method [36]. In brief, the mix of transformation plasmid and helper plasmid at a mole ratio 1:1 with a final concentration of 500 ng/µL was microinjected into the non-diapause silkworm embryos within 2 h after oviposition. The hatched G0 larvae were fed to oviposit the G1 eggs, and the positive G1 eggs were fluorescently screened through eyes where DsRed was specifically expressed by using an Olympus SZX12 fluorescence stereomicroscope (Olympus, Tokyo, Japan). A transgenic line was constructed by hybridizing the U6-gRNA and Nistari-Nos-Cas9 lines. BmMETTL4 mutant individuals with double fluorescence were used in subsequent experiments.

### 4.4. Genomic DNA Extraction and Mutagenesis Analysis

Genomic DNA was extracted from the epidermis shed in the prepupal stage and used for mutagenesis analysis. PCR amplification was performed to identify BmMETTL4 mutant alleles at the gRNA target site using primers in Table S1. PCR products were cloned into the pEASY^®^-Blunt Zero Cloning Vector (TransGen Biotech, Beijing, China) and sequenced (BGI, Shenzhen, China).

### 4.5. Quantitative Real-Time PCR Analysis

Total RNA was extracted from hatched silkworms of WT and unhatched from KO-METTL4 mutants on day 10 after egg laying by using a Total RNA Kit (Omega, Biel/Bienne, Switzerland). In total, 2 μg of each sample was used for reverse transcription to obtain the corresponding cDNA by a GoScript Reverse Transcription System (Promega, Madison, WI, USA). Subsequently, an equal amount of the cDNA was subjected for qRT-PCR assay using the SYBR Premix Ex TaqTM II kit (TaKaRa & Clontech, Dalian, China) on the Applied Biosystems 7500 Fast Real-Time PCR System (Applied Biosystems, Foster City, CA, USA). Eukaryotic translation initiation factor 4A (eIF-4a) was used as the internal control [37]. All experiments were independently performed with three biological replicates; the relative mRNA expression levels were calculated using the 2^−ΔΔCT^ method. All primers used for qRT-PCR were listed in Table S2.

### 4.6. Dot Blot Analysis

Dot blot experiment was performed according to the previous protocol [22]. In brief, genomic DNA samples were isolated hatched silkworms of WT and unhatched from KO-METTL4 mutants on day 10 after egg laying, and digested by RNaseA to rule out RNA contaminations. DNA were denatured at 95 °C for 5 min and spotted on the PVDF membrane (Roche, Welwyn Garden City, UK). The membranes were allowed to air dry and UV-crosslink (HL-2000 HybriLinker, Analytik Jena, Jena, Germany). Membranes were blocked and incubated with 6mA antibody (1:1000) (ABE572, Millipore, Billerica, MA, USA) overnight at 4 °C. After 3 washes for 10 min each, membranes were incubated with HRP linked secondary anti-rabbit IgG antibody (1:5000) (Beyotime, Shanghai, China) for 1 h at room temperature. Signals were detected by Thermo Fisher (Waltham, MA, USA) ECL reagent under a ChemiScope (CLiNX) Western blot processor.

### 4.7. RNA-Seq Analysis

Total RNA was extracted from hatched silkworms of WT and unhatched from KO-METTL4 mutants on day 10 after egg laying by using a Total RNA Kit (Omega, Biel/Bienne, Switzerland). For RNA-Seq, library construction and sequencing using a DNBSEQ platform was performed by BGI Company (Shenzhen, China). The raw sequencing data (Table S3) were qualified, filtered, and mapped to the reference silkworm genome database [38] by using bowtie2 [39]. The expression abundance of individual genes was determined as fragments per kb exon per million fragments mapped (FPKM). Differentially expressed gene (DEG) analysis was carried out by DEseq2 [40], with the standard of |log2 (Fold Change)| > 0 and *Padj* < 0.05. The DEGs were annotated functionally using Gene Ontology (GO) and Kyoto Encyclopedia of Genes and Genomes (KEGG) annotations. Principal component analysis (PCA) was a statistical procedure that used an orthogonal transformation to convert a set of observations of possibly correlated variables into a set of values of linearly uncorrelated variables. In these instances we used plotPCA function of DEseq2 program [40] to analyze the variables of FPKM counts from WT and KO-METTL4 samples. All the raw RNA-Seq data has been deposited in the NCBI with a BioProject accession number: PRJNA911404.

### 4.8. Photography and Scanning Electron Microscopy

Silkworms hatched from WT and unhatched from KO-METTL4 mutants on day 10 after egg laying were dissected and photographed under an EVOS FL Auto Microscope system (Life Technologies, Carlsbad, CA, USA). The surface of the silkworm heads was coated with Au (MCI000, Tokyo, Japan) and observed using scanning electron microscopy (SEM, Hitachi SU3500, Tokyo, Japan).

### 4.9. Statistical Analysis

All data were presented as the mean ± standard deviation (SD) of three independent biological replicates. Statistical significance (*p*-value) was analyzed by the Student’s *t*-test and denoted as follows: * *p* < 0.05, ** *p* <0.01, and *** *p* < 0.001.

## Data Availability

Data is contained within the article or Supplementary Materials.

## Supplementary Materials

The supporting information can be downloaded at: https://www.mdpi.com/article/10.3390/ijms24043468/s1.
